# Biodegradable membrane of poly(l-lactide acid-dioxanone-glycolide) and stereocomplex poly(lactide) with enhanced crystallization and biocompatibility

**DOI:** 10.3389/fbioe.2022.1021218

**Published:** 2022-09-30

**Authors:** Tiantang Fan, Jingwen Qin, Xiao Meng, Jiafeng Li, Qing Liu, Guannan Wang

**Affiliations:** ^1^ College of Medical Engineering & the Key Laboratory for Medical Functional Nanomaterials, Jining Medical University, Jining, China; ^2^ The Institute for Translational Nanomedicine, Shanghai East Hospital, The Institute for Biomedical Engineering & Nano Science, Tongji University School of Medicine, Shanghai, China; ^3^ College of Materials Science and Engineering, North China Electric Power University, Beijing, China; ^4^ China Coal Research Institute, Beijing, China

**Keywords:** poly(l-lactide), stereocomplex poly(lactic acid), crystallization, biodegradability, biocompatibility

## Abstract

The membranes of poly(l-lactide acid-p-dioxanone-glycolide) (PLPG) with stereocomplex poly(lactic acid) (sc-PLA) were prepared by the solution blending way. It was observed that sc-PLA significantly heightened the crystallizing behavior of PLLA segments of the PLPG matrix. The crystallizing behavior displayed that the temperature of crystallization shifted to a higher range than that of PLPG. Moreover, the half-time of crystallization sharply decreased in value as the sc-PLA content increased in value on account of the pre-eminent nucleation ability of sc-PLA. TGA results revealed the thermal stability of the samples with the increase of sc-PLA compared to PLPG. Meanwhile, enzymatic degradation results indicated that the mass loss rate of the membrane decreased with the introduction of sc-PLA, but the overall degradation ability was still greater than that of PLLA. In the meantime, the biological experiment indicated that the membrane possessed low cytotoxicity.

## 1 Introduction

Poly(l-lactide) (PLLA) has gained interest in tissue engineering and orthopedics fields owing to its mechanical properties, biodegradability, and easy processability (Liu et al., 2017; Ziemba et al., 2018; Shamsah et al., 2020). However, PLLA still has disadvantages, such as poor toughness and a slow degradation rate, that restrain its applications on a large scale (Vilay et al., 2011; Li et al., 2020a). Consequently, several researchers are trying to modify the structure for PLLA to enhance both properties simultaneously (Fasolino et al., 2017; Yang et al., 2018; Li et al., 2020b). Chemical modification methods are employed to strengthen the performance of polymers. Ring-opening polymerization has significant advantages, such as simple operation, mature technology, controllable product structure, and molecular weight, compared with the other chemical modification methods (Nikovia et al., 2019). Poly (p-dioxanone) (PPDO) has excellent mechanical strength, distinguished biodegradability, and biocompatibility, which are widely useful in surgical suturing, cardiovascular applications, and tissue engineering (Schattmann et al., 2017). Moreover, poly(lactic-co-glycolic acid) (PLGA) is another popular biomedical degradable polymer material, which can regulate the comprehensive properties by changing the ratio of LLA to GA and is widely employed in tissue engineering (Yoo and Won, 2020; Song et al., 2022). Hence, biodegradable PLLA-PDO-GA (PLPG) copolymers were prepared by ring-opening polymerization. Adding GA and PDO improves the performance of PLLA; however, GA and PDO interrupt the regularity of PLLA segments compromising the crystallization ability of the polymers. Consequently, it is indispensable to find out the enhancement mechanism to expand the practical applications of PLPG polymers.

Recently, several studies have reported blending PLLA with inorganic or organic materials to enhance the crystallization ability of PLLA (Chen et al., 2014; Tao et al., 2018; Purnama et al., 2021). Generally, the inorganic or organic materials reduce the energy barrier or surface-free energy barrier, thereby enhancing the crystallization kinetics of the materials. Wang and Qiu (2012) reported that the overall isothermal melt crystallization rates of PLLA/graphene oxide were greater than that of PLLA. Furthermore, Zhang et al. (2016) demonstrated that the talcum powder (Talc) and multiamide compound (TMC) could heighten the crystallizing ability of PLLA. However, the aforementioned inorganic materials still have defects such as easy migration and difficult degradation. Consequently, some organic materials are exploited to accelerate the crystallization ability of PLLA. Notably, stereocomplex poly(lactic acid) (sc-PLA) is extensively employed in biomedical fields due to its excellent mechanical properties and biocompatibility. Additionally, the melting temperature (T_m_) of sc-PLA is significantly higher than that of PLLA by 50°C; hence, the additional sc-PLA could remain in PLLA during its melting state. Previous studies have shown that sc-PLA could significantly enhance the crystallization ability of PLLA polymers. Furthermore, Fan et al. (2020) reported that sc-PLA would significantly strengthen the kinetic crystallizability of the PLLA segments in poly(TMC-b-(LLA-ran-GA)), and the crystallization half-time (t_0.5_) decreased from 12.3 to 3.7 min as sc-PLA increased from 3% to 20% at 120°C.

In the present study, a series of PLPG/sc-PLA (P/s) blends were prepared and a thermal process was designed to generate a “standard state” to examine the efficiency of nucleation. The influence of different sc-PLA content on the crystallization ability of PLLA and biocompatibility of PLPG copolymers was analyzed. Moreover, the enhanced mechanism was investigated to control the mechanical performances and biocompatibility of the PLPG polymers. We believe that the P/s membrane materials are expected to be used in skin trauma, bone tissue engineering, and so on.

## 2 Materials and methods

### 2.1 Materials

LLA, GA, and PDO were supplied by Daigang Biomaterial Co., Ltd. (China, >99%). Stannous octoate [Sn(Oct)_2_] was obtained from Adamas Reagent Co., Ltd. (Shanghai, China). Proteinase K with white powder was obtained from Solarbio (China). All the experiment reagents were used as received. Sc-PLA was prepared by the solution blending method according to the published literature (Fan et al., 2019). Briefly, PLLA was dissolved in dichloromethane (CH_2_Cl_2_). When PLLA was completely dissolved, PDLA was added to the aforementioned solution at a mass ratio of 50:50. After stirring for 6 h, the mixture solution was placed at room temperature. Finally, sc-PLA was obtained after the solution was completely volatilized.

### 2.2 Preparation of the PLPG copolymers

The PLPG polymers with 6.0 × 10^5^ g/mol and polydispersity values (PDI) of 1.38 were prepared by ROP. The molar ratio of LLA, GA, and PDO was set as 90: 5: 5 (Fan et al., 2020). Briefly, the right contents of LLA, GA, and PDO were put into a silanized tube using Sn(Oct)_2_. The tube was sintered under a vacuum after degassing and polymerization was accomplished at 135°C for 72 h. The obtained polymers were treated with CH_2_Cl_2_ and ethanol. Finally, the PLPG copolymers were dried at 50°C in a vacuum while keeping the mass constant. The yield of the PLPG copolymers was about 85%.

### 2.3 Preparation of the P/s membrane

The polymer membranes were prepared to employ the solution-casting method at room temperature. The various concentrations of sc-PLA in the samples were obtained by dissolving PLPG and sc-PLA solution of chloroform and 1,1,1,3,3,3-hexafluoro-2-propanol and stirred for about 6 h. Then, the solution was evaporated and the membrane was dried at 37°C in a vacuum drying oven for 24 h until the mass was constant. The weight ratio of sc-PLA to PLPG was 5, 10, 15, and 20 wt%, respectively. The membrane with χ wt% sc-PLA was named P/s-χ.

### 2.4 Characterizations

#### 2.4.1 Crystal structure and crystallization behavior

The X-ray diffraction (XRD) patterns of the samples were recorded by using the Bruker D8 advanced X-ray diffractometer with 40 kV and 25 mA. The diffractogram of the samples was gained from 5° to 35° using the Cu Kα radiation (4°/minute).

#### 2.4.2 Thermal behavior

DSC (Mettler Toledo, Switzerland) at N_2_ was used to display the thermal properties of the samples. Briefly, 4–8 mg of the samples was heated to about 230°C at 20°C/min first. Later, the samples were incubated for 3 min to clear off the thermal history and then cooled down to 20°C quickly. Finally, the sample was reheated to 230°C at 10°C/min to study the melting behavior. Furthermore, for non-isothermal crystallization, 4–8 mg of the membrane was heated to 230°C at 20°C/min and then maintained for 3 min. The temperature was dropped gradually to room temperature at 3°C/min. Additionally, the sample was heated to about 230°C and maintained for about 5 min. Then, the samples were cooled down to 155°C and kept for 10 min to generate sc-PLA completely. Finally, the samples were quickly quenched to 100, 105, 110, 115, 120, 125, and 130°C and held for about 30 min to examine the isothermal crystallization behavior of the samples.

#### 2.4.3 Enzymatic degradation

The square samples with dimensions 5 × 5 × 0.1 mm were analyzed for enzymatic degradation by treating them with protease K at different time intervals. Briefly, the samples were soaked in the proteinase K-tris buffer solution (3 ml, 0.05 M, pH = 8.5) and their weight before and after soaking was recorded. The activity of proteinase K was evaluated by changing the solution every 2 days. The samples were rinsed with distilled water three times at a scheduled time. In the end, the membranes were placed in the drying oven (37°C) until the mass was constant.

#### 2.4.4 Thermal degradation behavior

Thermogravimetric analysis (Hengjiu, Beijing) was employed to reveal the kinetics of thermal degradation. In brief, the membrane was heated to 500°C at 10, 15, 20, and 25°C/min, respectively.

#### 2.4.5 Cytotoxicity assay

The cytotoxicity of the samples was investigated on the human adipose-derived stem cells (hADSCs) using cell culture experiments. hADSCs were obtained from the GMP Laboratory of Stem Cell Transformation, medicine industry base (Shanghai East Hospital). The protocol for processing human tissues and cells was ratified by the Ethics Committee (Tongji University School of Medicine, Tongji University Affiliated East Hospital, and Jining Medical University). Adipose tissue donors signed informed consent and voluntarily donated samples. The viability was assessed by employing live/dead staining assay. Specifically, hADSCs (3 × 10^4^ cells/mL) were seeded into 96-cell culture plates that had leach liquors of the membrane at 37°C atmospheres under a medium (5% CO_2_). Furthermore, hADSCs were colored by calcein-AM and propidium iodide (PI) for 30 min after incubation for 1, 2, and 3 days. In the end, fluorescent staining was captured by using a fluorescence microscope (LEICA). The proliferation of hADSCs was quantified by the cell counting kit-8 (CCK-8) assays. hADSCs (3 × 10^4^ cells/mL) were cultured in 96-cell culture plates that contained the membrane leach liquor. Later, the CCK-8 solution was introduced into each well in the dark at the predetermined time. The absorbancy of the membrane was acquired by using a multimode reader at 450 nm.

The hADSCs grown in the leach liquor of the membrane were fixed with paraformaldehyde (4%) for about 10 min and permeated by the Triton X-100 solution (0.5%) for about 5 min. hADSCs were washed with PBS and colored to observe the actin cytoskeleton by the prepared RBITC-labeled phalloidin working reagents for about half hour in the dark conditions. Later, DAPI (5 μg/ml) was implied to detect the nuclear staining. Finally, fluorescent staining was performed by using the fluorescence microscope.

## 3 Results and discussion

### 3.1 Thermal properties and crystal structures

**FIGURE 1:**
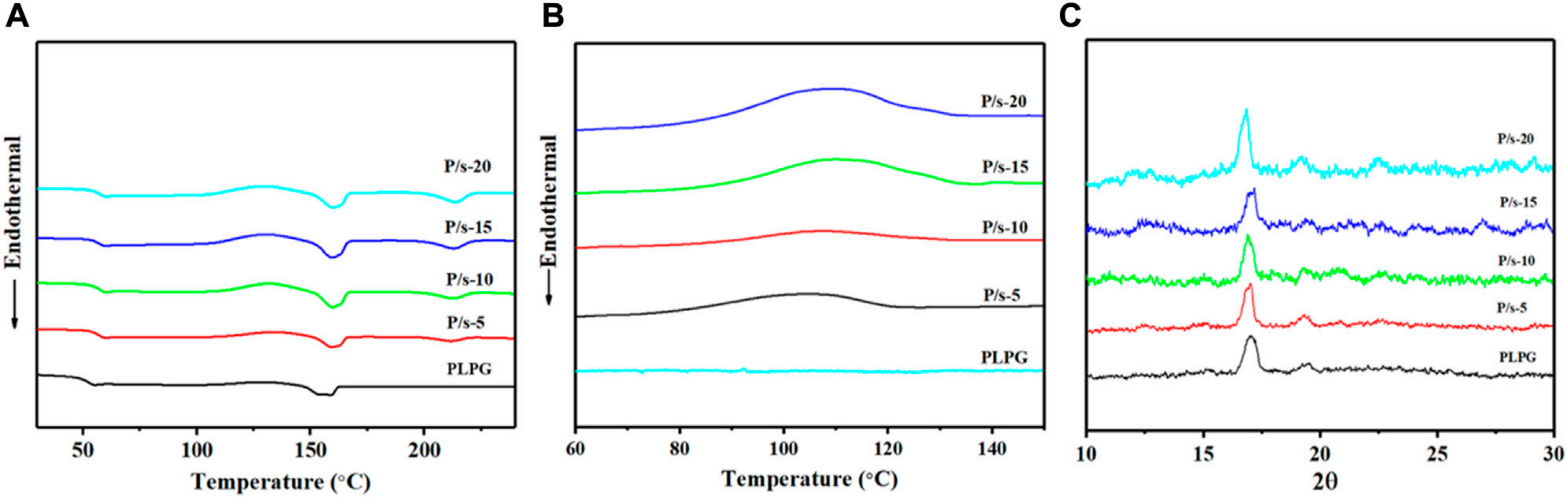
**(A)** Melting curves from 25°C to 230°C at 10°C/min. **(B)** Non-isothermal crystallization curves. **(C)** WAXD profile of the membrane at 3°C/min.

The crystallization behavior of polymer materials reflects their crystallization ability and the kinetics of the molecular chains in the polymers. Figure 1A shows the DSC curve of the second heating of the samples and after quenching from 230°C. Table 1 shows the thermal performance parameters such as cold crystallization temperature ( $T_{cc}$ ), melting point ( $T_{m}$ ), and melting enthalpy (∆ $H_{m}$ ). The gradual decrease in the temperature with the increase in the sc-PLA content was detected. Furthermore, $T_{cc}$ of the P/s-5 was 134.3°C; however, as the additional amount of sc-PLA reached 20 wt%, $T_{cc}$ of the P/s-20 decreased to 127.4°C. Meanwhile, a melting peak at about 159°C corresponding to $T_{m}$ of PLLA in the PLPG polymers was detected. Moreover, due to the addition of sc-PLA in the PLPG polymers, a new melting peak appeared at about 212°C, corresponding to $T_{m}$ of sc-PLA (Deng et al., 2021). Compared with the PLPG matrix, PLLA in the P/s was almost unchanged, while $∆H_{m-hc}$ increased gradually with the increase in the sc-PLA content. $∆H_{m-hc}$ of the PLPG matrix was 8.9 J/g with a corresponding crystallinity $\left(X_{c-hc}\right)\;o$ f 9.5%. $∆H_{m-hc}$ of the P/s-15 was 17.5 J/g with a corresponding $X_{c-hc}$ of 18.6%. $X_{c-hc}$ of the P/s-20 was slightly lower than that of P/s-15 (Table 1), suggesting that too much sc-PLA would hinder the migration of PLLA segments and weaken the crystallization ability of PLLA segments in the PLPG polymers. Meanwhile, $∆H_{m-sc}$ of sc-PLA of the P/s membrane increased gradually as the content of sc-PLA increased. $∆H_{m-sc}$ of sc-PLA in the P/s-5 was 2.8 J/g with a corresponding $X_{c-sc}$ of 1.8%. Furthermore, as the content of sc-PLA increased to 20 wt%, $∆H_{m-sc}$ of sc-PLA in the P/s-20 was 8.2 J/g, and $X_{c-sc}$ of sc-PLA was 5.3%. The aforementioned results indicated that adding sc-PLA can effectively improve the crystallization ability of PLLA segments of PLPG polymers.

**TABLE 1:** Thermal property parameters of the PLPG polymers and P/s membranes.

Membrane	$T_{cc}$ (°C)	$T_{m1}$ (°C)	*∆* $H_{m-hc}$ (J/g)	$X_{c-hc}$ (%)	$T_{m2}$ (°C)	*∆* $H_{m-cs}$ (J/g)	$X_{c-sc}$ (%)
PLPG	—	158.5	8.9	9.5	—	—	—
P/s-5	134.3	159.2	9.7	10.3	211.4	2.8	1.8
P/s-10	131.3	159.3	16.1	17.1	212.5	5.1	3.3
P/s-15	129.1	159.2	17.5	18.6	212.9	6.8	4.4
P/s-20	127.4	159.5	16.7	17.7	213.5	8.2	5.3

$T_{m1}$ and $T_{m2}$ are the melting points of PLLA and sc-PLA in the samples, respectively. $∆H_{m-hc}$ and $∆H_{m-sc}$ are the melting enthalpies of PLLA and sc-PLA in the samples, respectively. $X_{c-hc}$ and $X_{c-sc}$ are the crystallinity of PLLA and sc-PLA in the samples, respectively. $X_{c}$ =( $∆H_{m}$ / ${∆H}_{m}^{\infty }$ ) × 100%, and ${∆H}_{m}^{\infty }$ of PLLA and sc-PLA are 94 J/g and 155 J/g (Li et al., 2016), respectively.

Furthermore, the non-isothermal crystallization behavior was tested to assess the impact of sc-PLA in enhancing kinetic crystallizability of the PLLA segments of PLPG polymers. Figure 1B shows crystallization temperature ( $T_{c}$ ), crystallization enthalpy ( $∆H_{c}$ ), and crystallinity ( $X_{c}$ ) at the cooling rate of 3°C/min. The crystallization and melting behavior of the samples exhibited dependence on the sc-PLA content in the polymer. Moreover, the crystallization peak of PLLA segments in PLPG polymers cannot be detected, suggesting that the crystallization ability of PLLA was negligible at the cooling rate of 3°C/min. Conversely, the crystallization peak was detected with the introduction of sc-PLA, and $T_{c}$ shifted to a higher temperature with increased sc-PLA. The results implied that the induction of sc-PLA strengthened the crystallization ability of PLLA segments of PLPG polymers. The results suggested that sc-PLA provided enough heterogeneous nucleation sites and reduced the energy barrier for nucleation, thus heightening the kinetic crystallizability of PLLA segments of PLPG polymers.

Additionally, the crystal structures of the samples that cooled from 230 to 25°C at 3°C/min were analyzed by XRD (Figure 1C), and the crystallization peaks of PLLA and sc-PLA in the blends were detected. The crystallization peaks at about 14.7°, 16.8°, 19.3°, and 22.4° could be assigned to the crystals of PLLA of PLPG polymers corresponding to the (010), (200)/(110), (203), and (210) crystal grids, respectively (Jiang et al., 2015; Park and Hong, 2021). The crystallization peaks of sc-PLA were 11.8°, 20.6°, and 24°. The aforementioned results displayed that adding sc-PLA would effectively heighten the crystallization ability of the PLLA segment of PLPG polymers.

**FIGURE 2:**
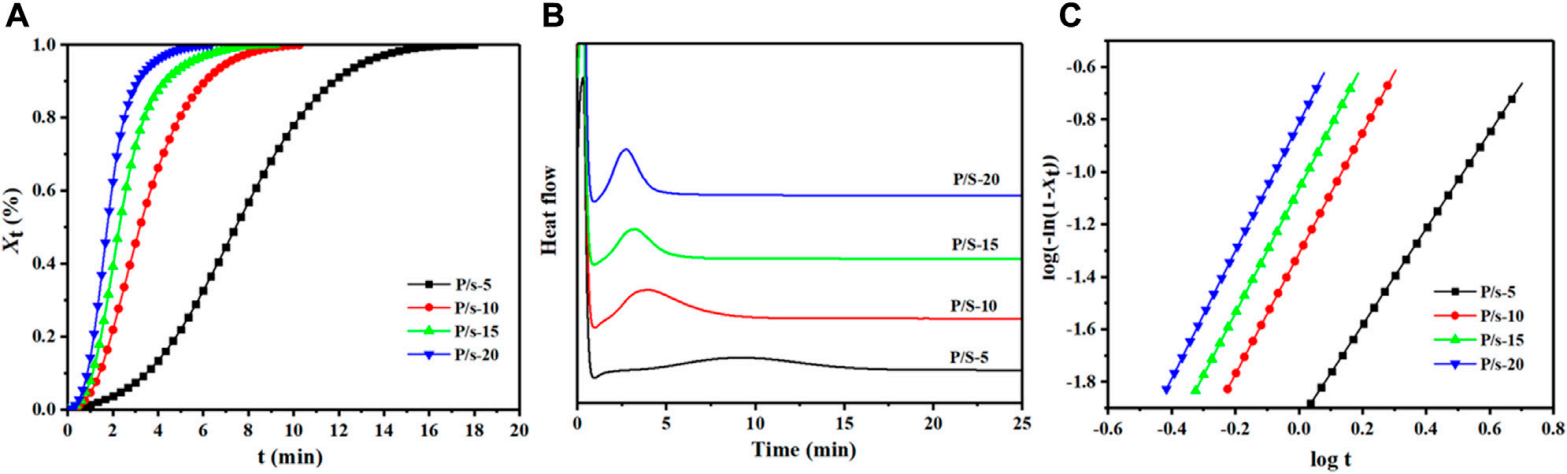
**(A)** DSC heat flow at 125°C, **(B)** *X*_t_ from DSC at 125°C, and **(C)** plots of log[-ln (1- $X_{t}$ )] *versus* log *t* at 125°C.

The isothermal crystallization was measured by DSC, and the enhancement impact of adding sc-PLA on the crystallization ability of PLLA segments of PLPG polymers was evaluated (Figure 2). $T_{c}$ of the isothermal crystallization was selected from 100°C to 125°C. The relative degree of crystallinity ( $X_{t}$ ) was calculated by the following equation (Cartier et al., 1997):

$$X_{t}=\frac{\overset{t}{\underset{0}{\int }}\left(\frac{dH}{dt}\right)dt}{\overset{\infty }{\underset{0}{\int }}\left(\frac{dH}{dt}\right)dt}=\frac{∆H_{t}}{∆H_{\infty }}.$$

Here dH/dt was the enthalpy change rate, $∆H_{t}$ was the heat enthalpy, and $∆H_{\infty }$ was the total heat enthalpy calculated. A broad exothermal peak for the samples with lower sc-PLA content was detected, suggesting an influence of the content of sc-PLA on crystallization (Figures 2A,B). Furthermore, with an increase in the sc-PLA content, the exothermal peaks became narrow by degrees and $X_{t}$ decreased, indicating a strengthening of the crystallization ability. The crystallinity curves were of S-shape, and there was an obvious induction period in the early stage. In addition, with the increase in the sc-PLA content, the crystallinity conversion rate compared with the initial stage increased, suggesting that sc-PLA could be defined as a heterogeneous nucleation site to reduce the free energy barrier. Furthermore, as the content of sc-PLA increased, the number of nucleation sites was increased, resulting in an enhanced crystallization rate of PLLA segments of the P/s membranes. These results indicated that sc-PLA had an obvious enhancing impact on the overall crystallization rate of PLLA segments of PLPG polymers. t_0.5_ was calculated at $X_{t}$ = 50% (Supplementary Table S1). t_0.5_ decreased with the introduction of sc-PLA as $T_{c}$ was below 120°C, which could be due to a lower subcooling degree and more flexibility in the molecular chain movement, leading to a hard arrangement in the lattice. In the P/s-5, t_0.5_ decreased from 10.8 to 5.2 min, which corresponded to $T_{c}$ of 100°C and 115°C, respectively. As $T_{c}$ was above 120°C, t_0.5_ increased from 5.2 min to 7.4 min. Meanwhile, as the content of sc-PLA increased, t_0.5_ decreased under the same $T_{c}$ . These results showed that the introduction of sc-PLA could significantly enhance the crystallization ability of PLLA segments in PLPG polymers. In addition, the isothermal crystallization process of the P/s samples was also calculated by the Avrami equation (Lu et al., 2021).

$$\log\left[-\ln\left(1-X_{t}\right)\right]=\log k+n\;\log t.$$

Here n was the Avrami index and k was the overall crystallization rate. n and k can be acquired by plotting log[−ln (1- $X_{t}$ )] versus log t, which is the slope and intercept, respectively. Lorenzo et al. pointed out that $X_{t}$ (3%–20%) was selected to evaluate the values of n and k (Arnaldo et al., 2007). The overall crystallization kinetics in the primary crystallization range was entirely denoted using the Avrami equation (Figure 2C). The n values of P/s samples oscillated between 2.0 and 3.0 (Supplementary Table S1), corresponding to the crystal growth mechanism of three-dimensional spherulites.

### 3.2 Thermal degradation behavior

**FIGURE 3:**
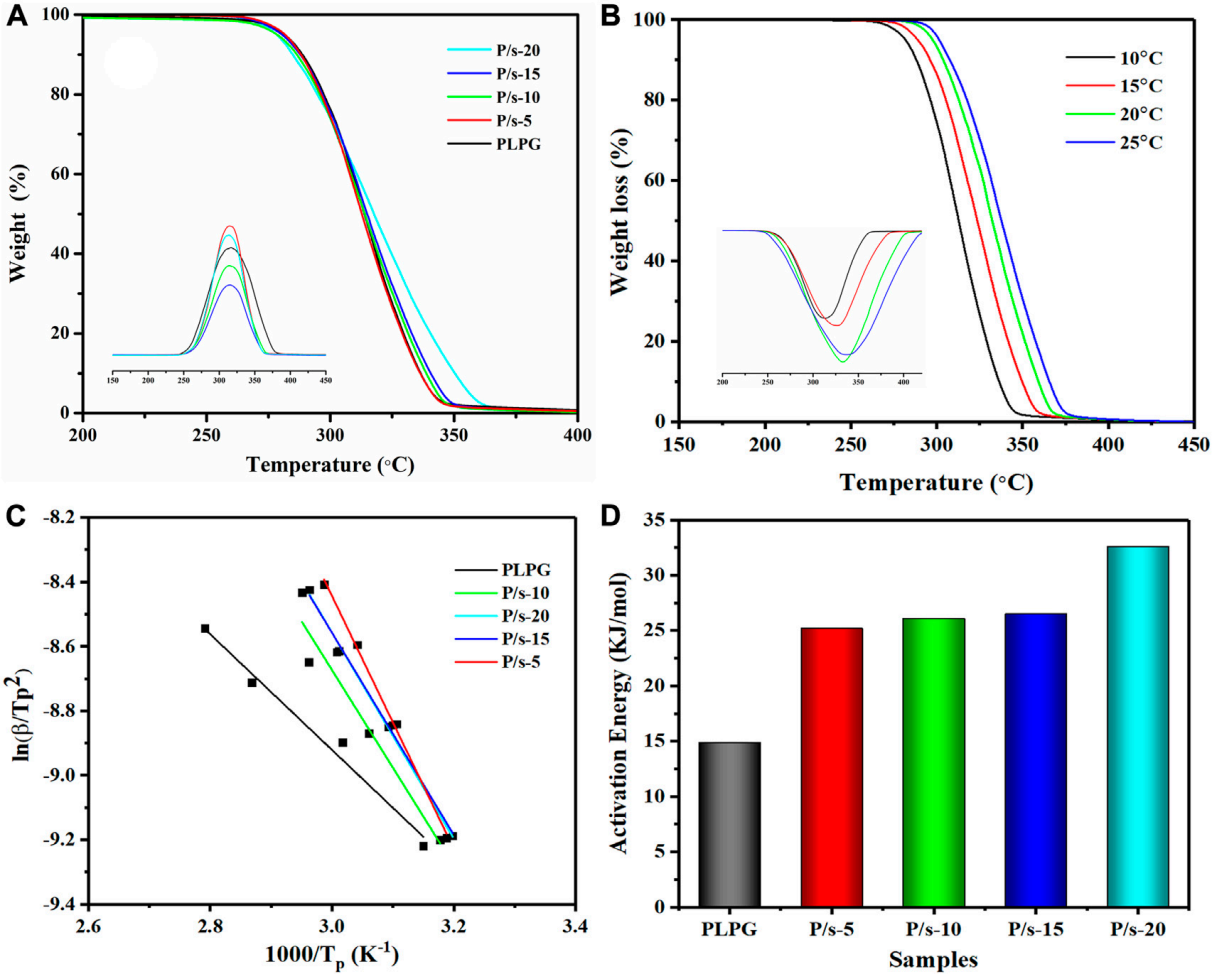
**(A)** TGA and DTG of the P/s membrane at 10°C/min, **(B)** TGA and DTG of the P/s-20 at 10, 15, 20, and 25°C/min, **(C)** Kissinger method that was applied to the experimental data, and **(D)** apparent $E_{a}$ data.

The thermal degradation behavior of P/s membranes influenced by adding sc-PLA was examined by TGA at 10°C/min (Figure 3A). All the samples were observed to be stable below 200°C, and single-stage thermal degradation could be detected as the heating temperature reached 400°C. Meanwhile, adding sc-PLA enhanced the thermostability of the P/s membranes compared to that of PLPG copolymers. The P/s-20 had better thermal stability than the other membranes, and the temperature of the maximum mass loss rate ( $T_{P}$ ) was about 314°C. This may be due to the following reasons. First, the thermal stability of sc-PLA was higher than that of PLLA. Meanwhile, the PLLA segments in PLPG and PDLA segments in sc-PLA could form the stereocomplex effect, which made the molecular chain bind closer and enhanced the intermolecular force.

At the same time, the Kissinger method was applied to analyze the kinetics of thermal degradation of the P/s membrane (Figure 3B). The TGA plots moved to the higher temperature region with a heating rate up to 25°C due to the requirement of the specific temperature in a shorter time and release gaseous products faster. The values are calculated using the following equation (Monika and Katiyar, 2017):

$$\ln\frac{\beta }{T_{P}^{2}}=-\frac{E_{a}}{R}*\frac{1}{T_{P}}+\ln\frac{AR}{E}.$$

Here, β was the heating rate. A was the pre-exponential factor. R was the gas constant. Figures 3C,D show the smooth linear fitted lines, indicating that the kinetics of thermal decomposition could be described by the Kissinger method. Furthermore, thermal degradation activation energy ( $E_{a}$ ) gradually increased with the increase of the sc-PLA content. $E_{a}$ of PLPG was about 14.9 kJ/mol. When the content of sc-PLA was increased to 20 wt%, $E_{a}$ of P/s-20 increased to about 32.6 kJ/mol (Figure 3D). This is due to the higher thermal stability than that of PLLA. Further addition of sc-PLA accelerated the crystallization ability of PLLA of PLPG polymers, thus leading to the higher PLLA content in the P/s membranes.

### 3.3 Preparation and enhancement mechanism

**SCHEME 1:**
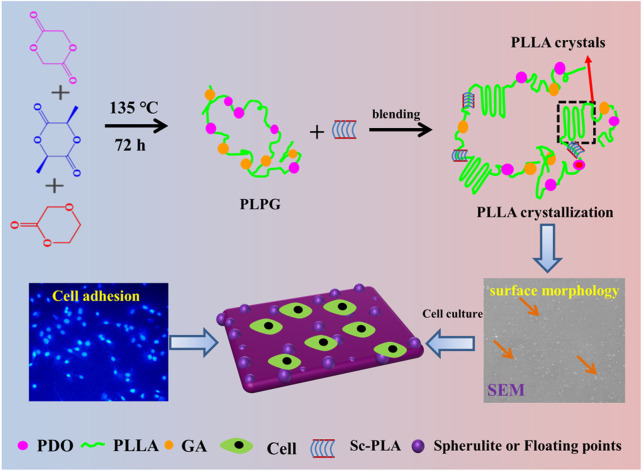
Schematic illustration of the preparation and enhancement mechanism of crystallization and improvement of cell adhesion of the P/s membranes used as biodegradable materials.

A series of controlled P/s membranes was prepared using the solution blending methods (Scheme 1). Epitaxial nucleation and chemical mechanisms are often applied to explain the enhancement of nucleating materials (Hall et al., 2014; Xu et al., 2022). The epitaxial crystallization of polymers that grow on organic materials is usually applied to manifest the crystallization behaviors of PLLA (Takenaka et al., 2004). Meanwhile, sc-PLA and the PLPG matrix have similar crystal structures, and sc-PLA heightened the crystallization of the second crystalline phase by weakening the free energy of activation (Scheme 1). With the increase of sc-PLA contents, more spherical crystals appeared on the surface of P/s membranes, thus generating many floating points, which were beneficial to improve the ability of cell adhesion and growth. So the P/s samples would possess good biocompatibility and biodegradability.

### 3.4 Enzymatic degradation behavior

**FIGURE 4:**
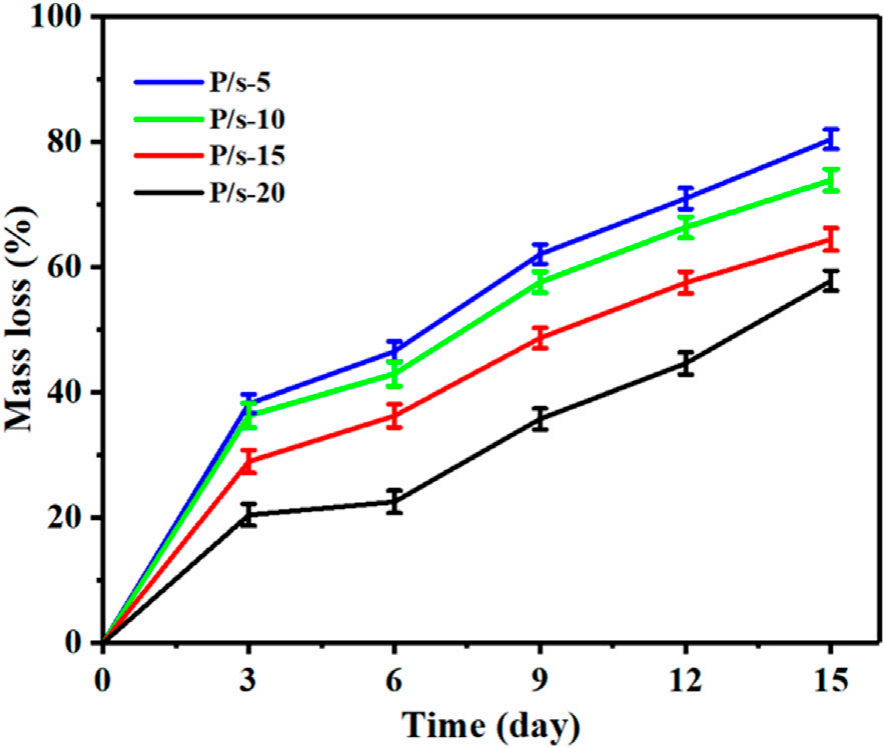
Mass loss of the membrane by enzymatic degradation for 15 days.

Enzyme degradation performance is an important index for evaluating completely degradable biomedical polymer materials. Figure 4 shows the influence of adding sc-PLA on the degradation performance of the P/s membrane by the protease K degradation experiment. The mass loss of the samples was calculated by the following equation:

$$Mass\;loss\;\left(\%\right)=\frac{\left(M_{i}-M_{d}\right)}{M_{i}}*100.$$

Here, $M_{i}$ and $M_{d}$ were the initial weight and dry weight of the membrane, respectively.

It can be observed that the mass loss of the samples showed a linear trend of increase with the extension of the enzyme degradation time (Figure 4). The samples showed a linear trend of increase with the extension of the enzyme degradation time. Moreover, with the increase of sc-PLA contents, the mass loss of the P/s membrane decreased gradually. The mass loss of the P/s-15 and P/s-20 membranes was greatly reduced and calculated to be 64.45% and 57.84%, respectively. Only the amorphous regions of PLLA could be degraded by proteinase K. Adding sc-PLA enhanced the crystallization ability of PLLA segments of PLPG polymers, thus improving X_c_ of PLPG polymers. Furthermore, since the stereocomplex structure is not easily degraded by proteinase K, the enzymatic degradation of sc-PLA was less efficient than that of PLLA. Therefore, with the increase of sc-PLA content, the mass loss of the P/s membrane decreased.

**FIGURE 5:**
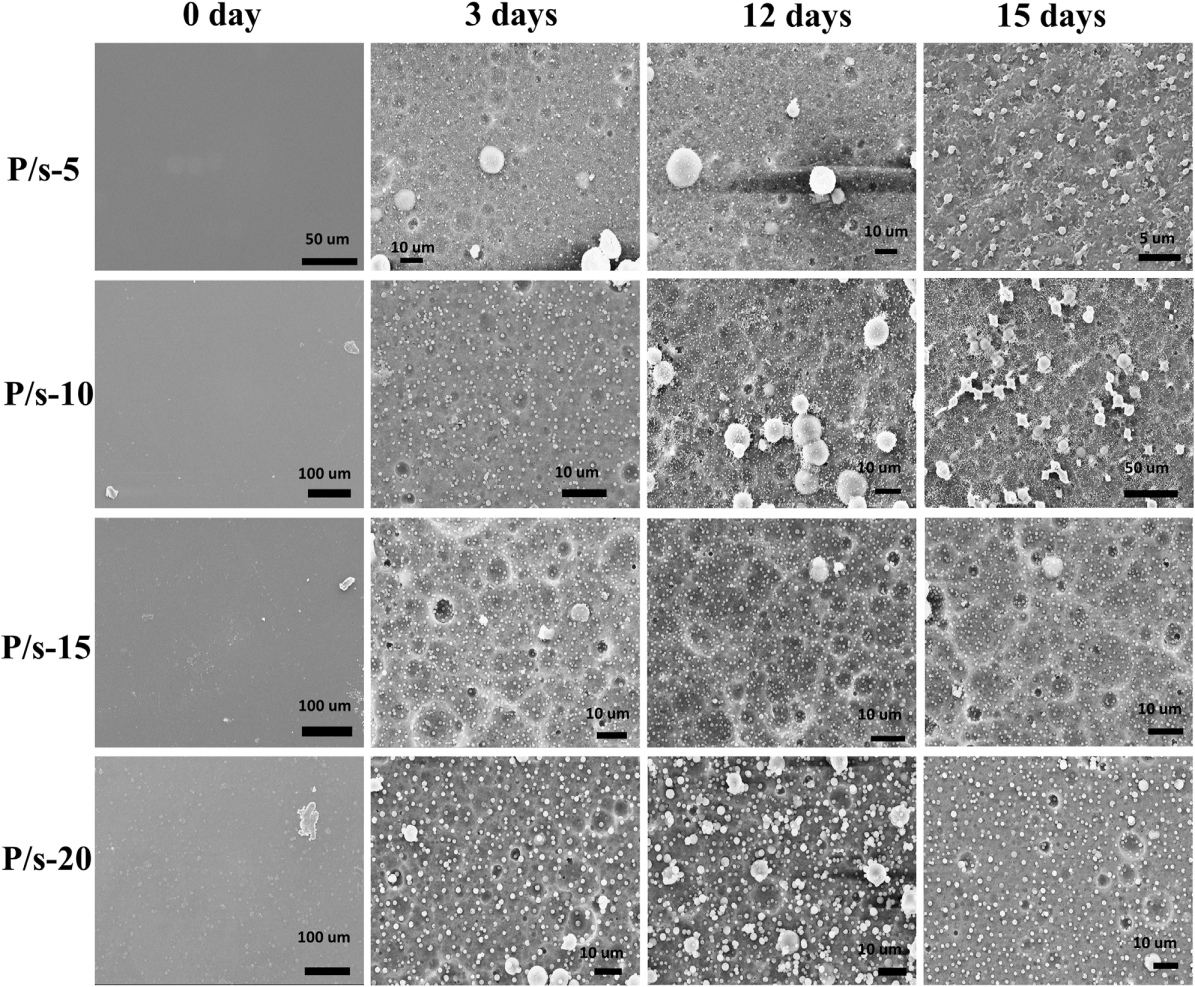
Surface morphology of the membranes during enzymatic degradation for 15 days.

Generally, the protease K degradation mechanism follows surface erosion degradation, and protease K has a high degree of selectivity to the degraded materials (Reeve et al., 1994). Moreover, protease K can degrade the amorphous region but not the crystalline region of PLLA materials. This is mainly due to the complex and larger structure of protease K, which cannot enter the interior of the polymer material. Therefore, protease K could only be adsorbed over the surface of the polymer material, causing breakage of the molecular chain on the polymer surface, and finally exposing the interiors of the polymer material for further degradation. The changes in the surface morphology of the samples during enzymatic degradation after 15 days were detected (Figure 5). The surface of the initial membrane was smooth, and a few irregular spherulites were observed on the surface of the membrane. When the degradation time lasted 3 days, the amorphous areas of the membranes were gradually eroded by the action of protease K. Hence, the surface appeared uneven with a little of pits. Furthermore, the amorphous region around the spherulite was eroded by protease K, while the spherulite could not be degraded and gradually fell off the surface during the degradation process. The increase in the degradation time of protease K to 15 days resulted in a large number of spherulites, and almost no amorphous areas on the surface of the membrane was detected. The enzymatic degradation after 15 days showed further reduction in the amorphous area and increased appearance of granular structures on the surface of the samples.

### 3.5 *In vitro* cellular assay

**FIGURE 6:**
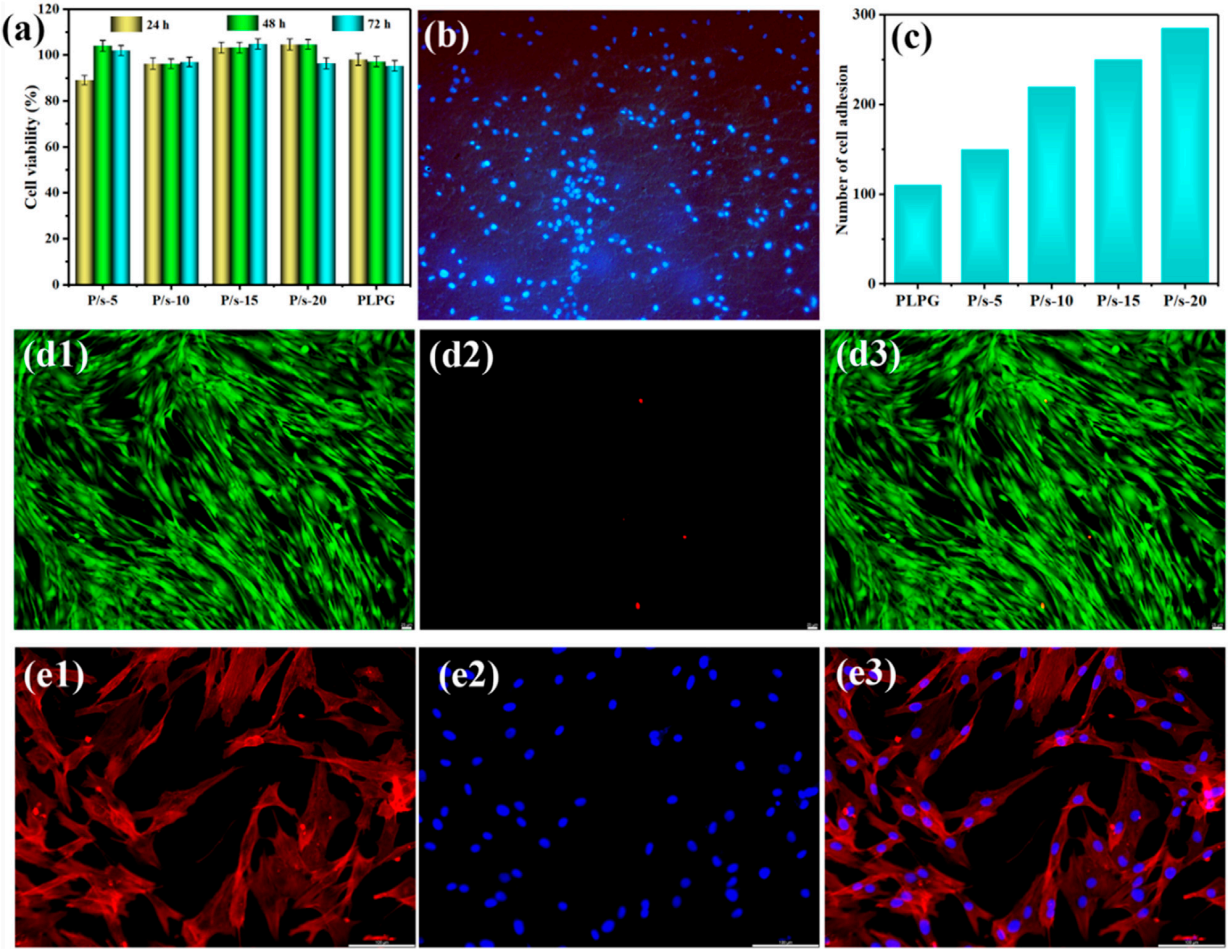
**(A)** CCK-8 assay of hADSCs for 24, 48, and 72 h, **(B)** cell adhesion on the surface of P/s-10 for 4 h ( × 10), **(C)** number of cell adhesions on the surface of the samples for 4 h, **(d1–d3)** live/dead cell viability of the P/s-10 blends for 48 h ( × 20), and **(e1–e3)** representative immunofluorescence microscopy images of the P/s-10 blends for 48 h ( × 20).

Figure 6 shows the cytotoxicity of hADSCs co-cultured with the samples. The cell viabilities of the samples analyzed by CCK-8 were all above 90%, and the cell viability increased with the extension of culture time to 72 h (Figure 6A). The cells could better adhere to the surface after being co-cultured with the P/s-10 membranes for 4 h (Figure 6B). Moreover, the number of cell adhesions increased by degrees as the sc-PLA content increased (Figure 6C).

The cytotoxicity of membranes was examined by live/dead staining (Figure 6d1–d3). The green and red fluorescences were the live cells and dead cells, respectively. All the membranes cultured for 48 h had low cytotoxicity with few dead cells (Figure 6d1–d3). Furthermore, the cell nucleus was stained with red color and the cytoplasm was stained with blue color for immunofluorescence microscopy images. It can be attributed that with the increase of sc-PLA, the surface of P/s membranes gradually became rough (Figure 4), which was consistent with the published literature (Li et al., 2020b). Figure 6(e1–e3) shows that the hADSCs co-cultured with the sample extract were well-distributed in shape and slender filamentous pseudopods, while the large number of straight actin stress fibers were observed to be well organized. Therefore, the aforementioned results revealed that the P/s blends possessed good biocompatibility.

## 4 Conclusion

Sc-PLA showed a dramatically improving effect on the crystallization ability of PLLA segments of PLPG polymers. The non-isothermal and isothermal crystallization results indicated that the crystallization behavior of the membranes obviously heightened owing to the nucleation density, and the crystallization acceleration became more prominent. The enzymatic degradation behavior showed that as the sc-PLA content increased, the weight loss of the membranes decreased by degrees and was still greater than that of PLLA polymers. Meanwhile, the biological experiments indicated that the P/s membranes had good cytocompatibility. Therefore, the aforementioned results demonstrated a significant enhancing impact of sc-PLA on the crystallization behavior of PLLA segments of PLPG polymers, and the P/s membranes possessed good cytocompatibility.

## Data Availability

The original contributions presented in the study are included in the article/Supplementary Material; further inquiries can be directed to the corresponding authors.
