# Supplementary material for: Biodegradable membrane of poly(l-lactide acid-dioxanone-glycolide) and stereocomplex poly(lactide) with enhanced crystallization and biocompatibility
Source: Front Bioeng Biotechnol. 2022 Sep 30;10:1021218. doi: 10.3389/fbioe.2022.1021218 (PMC9561826; doi:10.3389/fbioe.2022.1021218)
Supplement: Supplementary file 1 [file Table1.docx]

**Table S1.** The overall crystallization kinetic parameters of the P/s blends at 100^o^C, 105^o^C, 110^o^C, 115^o^C, 120^o^C and 125^o^C.

| Samples | $T_{c}$(^o^C) | *t*_0.5_ (min) | *n* | *k* (min^-n^) |
| --- | --- | --- | --- | --- |
| P/s-5 | 100 | 10.8 | 2.54 | 1.29 × 10^-2^ |
|  | 105 | 6.8 | 2.68 | 2.10 × 10^-2^ |
|  | 110 | 5.3 | 2.79 | 2.55 × 10^-2^ |
|  | 115 | 5.2 | 2.00 | 1.99× 10^-2^ |
|  | 120 | 5.3 | 2.66 | 2.73× 10^-2^ |
|  | 125 | 7.4 | 2.84 | 1.11 × 10^-2^ |
| P/s-10 | 100 | 7.1 | 2.66 | 2.23 × 10^-2^ |
|  | 105 | 2.5 | 2.06 | 1.30 × 10^-1^ |
|  | 110 | 1.8 | 2.21 | 2.16 × 10^-1^ |
|  | 115 | 5.3 | 2.78 | 2.64× 10^-1^ |
|  | 120 | 4.8 | 2.72 | 3.71× 10^-2^ |
|  | 125 | 3.2 | 2.30 | 8.61 × 10^-2^ |
| P/s-15 | 100 | 1.8 | 2.14 | 1.90 × 10^-1^ |
|  | 105 | 1.2 | 2.19 | 4.30 × 10^-1^ |
|  | 110 | 1.4 | 2.33 | 2.85 × 10^-1^ |
|  | 115 | 1.5 | 2.53 | 2.64× 10^-1^ |
|  | 120 | 1.8 | 2.49 | 1.55× 10^-1^ |
|  | 125 | 2.2 | 2.37 | 8.62 × 10^-2^ |
| P/s-20 | 100 | 1.5 | 2.14 | 2.51 × 10^-1^ |
|  | 105 | 1.0 | 2.44 | 6.33 × 10^-1^ |
|  | 110 | 1.5 | 2.34 | 3.16× 10^-1^ |
|  | 115 | 1.3 | 2.45 | 3.23× 10^-1^ |
|  | 120 | 1.5 | 2.27 | 2.65 × 10^-1^ |
|  | 125 | 1.7 | 2.43 | 1.53 × 10^-1^ |
